# Allorecognition behaviors in Myxomycetes respond to intraspecies factors

**DOI:** 10.1242/bio.060358

**Published:** 2024-07-25

**Authors:** Mana Masui, Phillip K. Yamamoto, Nobuaki Kono

**Affiliations:** ^1^ Faculty of Environment and Information Studies, Keio University, Fujisawa, Kanagawa, 252-0882, Japan; ^2^ Institute for Advanced Biosciences, Keio University, Tsuruoka, Yamagata, 997-0017, Japan; ^3^ Graduate School of Media and Governance, Keio University, Fujisawa, Kanagawa 252-0882, Japan

**Keywords:** Slime mold, Myxomycetes, Plasmodium, Allorecognition

## Abstract

Myxomycetes are multinucleate unicellular organisms. They form a Plasmodium that moves by protoplasmic flow and prey on microorganisms. When encountering intraspecifics, the plasmodium has the capacity for ‘fusion’, actively approaching and fusing its cells, or ‘avoidance’, altering its direction to avoid the other individual. This is an allorecognition ability. However, it remains unclear whether the range of allorecognition extends to other species, and its ecological significance is also obscure. Here, we conducted a quantitative evaluation of contact responses from closely related species of plasmodium to clarify the range of allorecognition behaviors in Myxomycetes. Behavioral assays demonstrated that allorecognition behaviors are specifically observed within individuals of the same species, indicating that these behaviors are a phenomenon unique to intraspecies interactions. Myxomycetes allorecognition is an extremely narrow and inward-focused behavior, suggesting a highly specialized mechanism.

## Teaser

**Summary:** Myxomycetes plasmodium can recognize one another only if they are of the same species.

## INTRODUCTION

Myxomycetes are multinucleate unicellular organisms belonging to the supergroup Amoebozoa (Adl et al., 2012; Keeling et al., 2005; Walker and Stephenson, 2016). They possess up to 800,000 nuclei per 1 mm^3^ (Gray and Alexopoulos, 1968). Approximately 1200 Myxomycetes species have been described worldwide (Lado, 2016), and these species exhibit various morphological forms throughout their life cycle, including the trophic form, fruiting body, and slime mold amoeba (Clark and Haskins, 2015; Gray and Alexopoulos, 1968). Nutrient uptake in plasmodium is accompanied by migration under the action of protoplasmic flow at a rate of 1 mm/s, a process driven by the cytoskeletal elements of actin and myosin (Kamiya, 1950). The primary nutritional sources, comprising chiefly bacteria and fungi, are assimilated via endocytosis (Chestnut, 1985).

The cellular growth strategy during the plasmodial phase is notably intriguing. Beyond growth via synchronous nuclear division occurring in consistent cycles (Gray and Alexopoulos, 1968), plasmodia also employ a strategy of fusion with other individuals of the same species (Hsu and Schaposnik, 2022; Jeffery and Rusch, 1974; Rusch et al., 1966). This phenomenon has been reported in the genera *Physarum* and *Didymium* (Carlile and Dee, 1967; Collins, 1966).

**Fig. 1.**
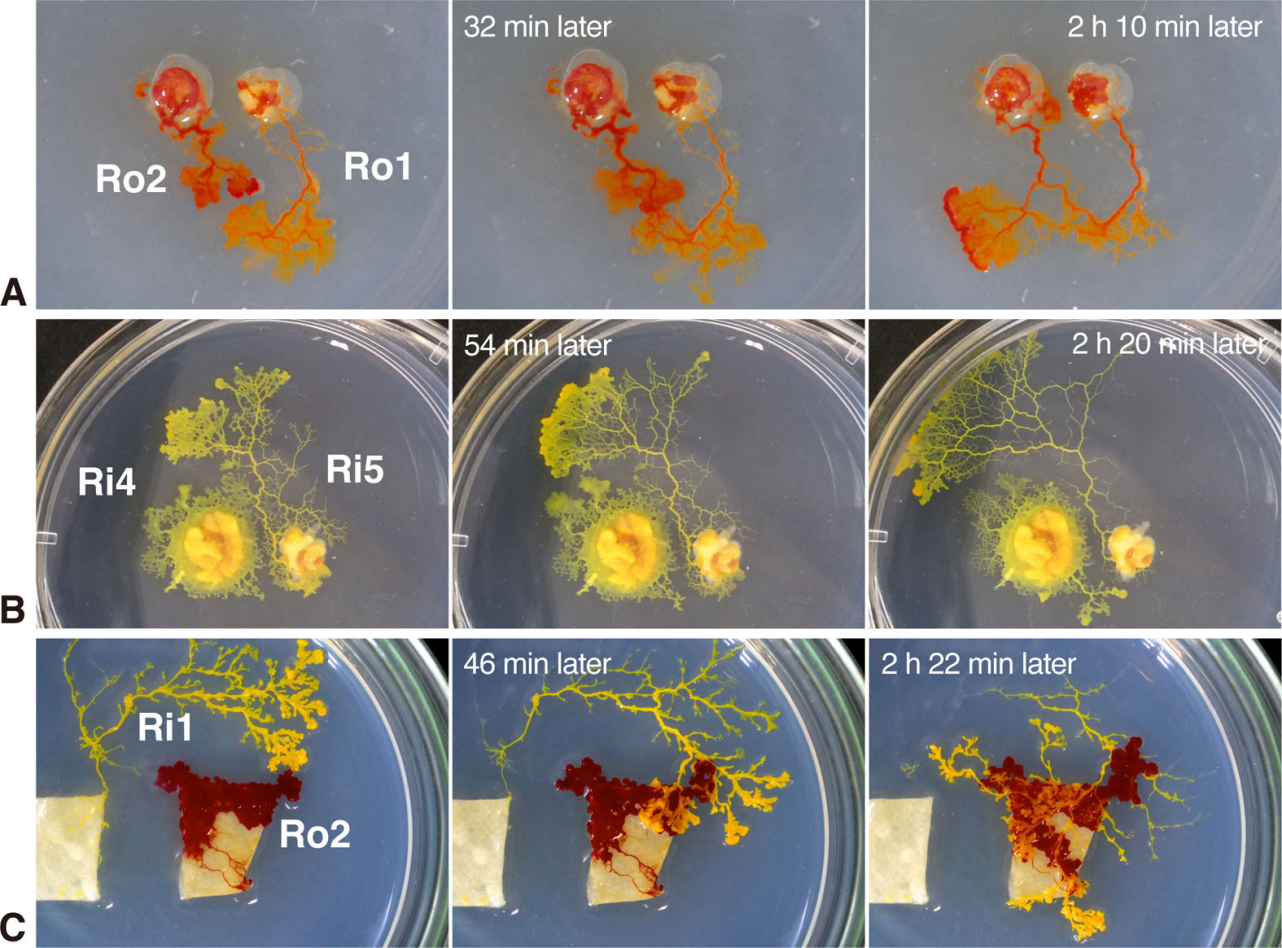
**Images demonstrating typical behavioral examples.** (A) Fusion behavior: after two strains of *Physarum roseum* (Ro2 and Ro1) encounter each other, they fuse with each other and become one individual. (B) Avoidance behavior: after two strains of *Physarum roseum* (Ri4 and Ri5) came into contact with each other, Ri5 changed the direction of movement and avoided fusion. (C) Riding-over behavior: yellow *P. rigidum* (strain Ri1) riding over red *P. roseum* (strain Ro2) without showing any reaction.

Fusion among plasmodia is not mediated by nuclear fusion as in sexual reproduction, but is a phenomenon of sharing cytoplasm while retaining individual nuclei (Rusch et al., 1966). Fusion is not universally feasible across all plasmodial relations. Upon interspecific encounters, plasmodia recognize intraspecifics through ‘allorecognition’ and facilitate a choice between ‘fusion’, in which they actively approach and fuse, and ‘avoidance’, in which they change direction to avoid the other individual (Fig. 1A,B) (Masui et al., 2018).

The genetic loci implicated in this selective process have been hypothesized, although their precise roles remain obscure (Clark, 2012; Collins and Haskins, 1972; Ling and Clark, 1981). The ecological relevance of such allorecognition behavior remains to be fully understood. In related studies, a cellular slime mold that shares a close evolutionary relationship with Myxomycetes was identified. Cellular slime molds are known to gather amoebae of the same species to form reproductive structures, such as fruiting bodies and spores, which use chemical signals to attract other species (Bonner et al., 1972). The formation of fruiting bodies occurs only in amoeboid cells of the same genetic lineage (Nicol and Garrod, 1978). This suggests that the allorecognition behavior of cellular slime molds helps them to find intraspecific information, a critical factor for the survival of their offspring.

Ants, as eusocial insects, can recognize their nest mates through cuticular hydrocarbons. This mechanism can also enable the identification of different species and nonnest mates of the same species (Iwai et al., 2022; Shelby and Deborah, 2012; Torres et al., 2007). This recognition behavior in ants contributes to the survival of the colony as a whole by discriminating between the elimination of external enemies and the communication of information with nest mates.

Considering the examples described thus far, the following hypothesis can be posited regarding the ecological role of allorecognition. Should allorecognition behaviors encompass Myxomycetes broadly, extending to interspecies recognition, such behaviors might function as a strategy for mitigating competition, for instance, in nutritional aspects, with individuals beyond the fusion capability. Conversely, if these behaviors are confined to intraspecies recognition, they would be insufficient to circumvent competition from other plasmodia. Hence, these behaviors could be construed as optimally designed for searching for fusible individuals.

Here, we aimed to determine the range over which allorecognition behaviors can be observed and their ecological significance by examining how Myxomycetes plasmodium responds to closely related species. Behavioral tests have utilized *Physarum ridium*, a Myxomycetes that has already been established for recognizing intraspecific plasmodium (Collins, 1966; Masui et al., 2018). Although *P. ridium* has been shown to engage in fusion and avoidance behaviors within the same species, its reactions to different species remain unexplored. In particular, a quantitative evaluation of behaviors between different species has not been conducted. By performing such an evaluation, we can expect to demonstrate intraspecific and interspecific species-specific behaviors using quantitative data. Against this background, *Physarum roseum*, a different species within the same genus as *P. rigidum*, is introduced. By observing encounters between *P. rigidum* and *P. roseum*, we investigated how far allorecognition adapts, exploring its range of responses. It is also possible that an exclusive behavior when they come into contact may be observed. This finding was also confirmed through behavioral tests.

## RESULTS

### Species identification

**Fig. 2.**
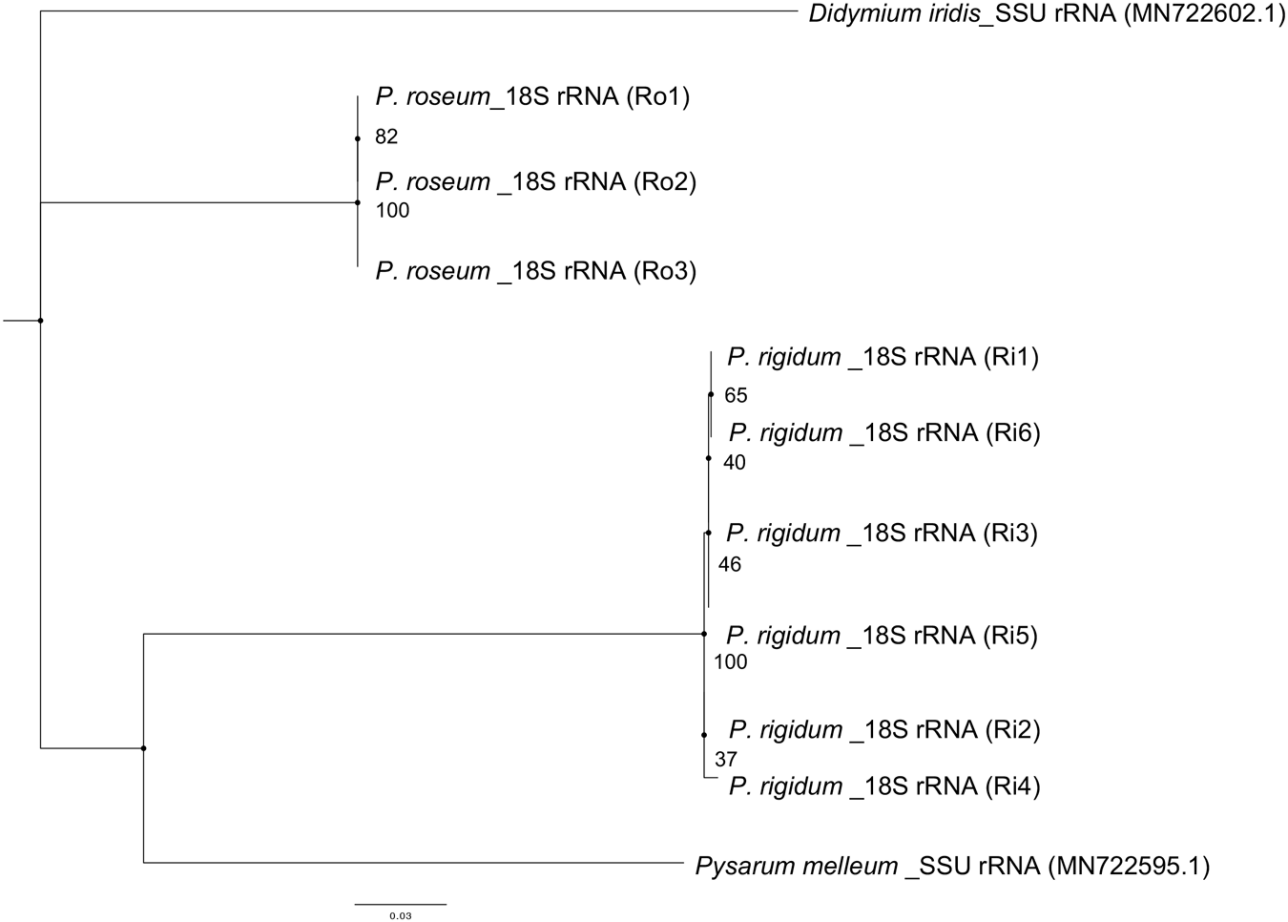
**Phylogenetic tree of the 18S rRNA region.** Ri strains and Ro strains formed different clades.

**Fig. 3.**
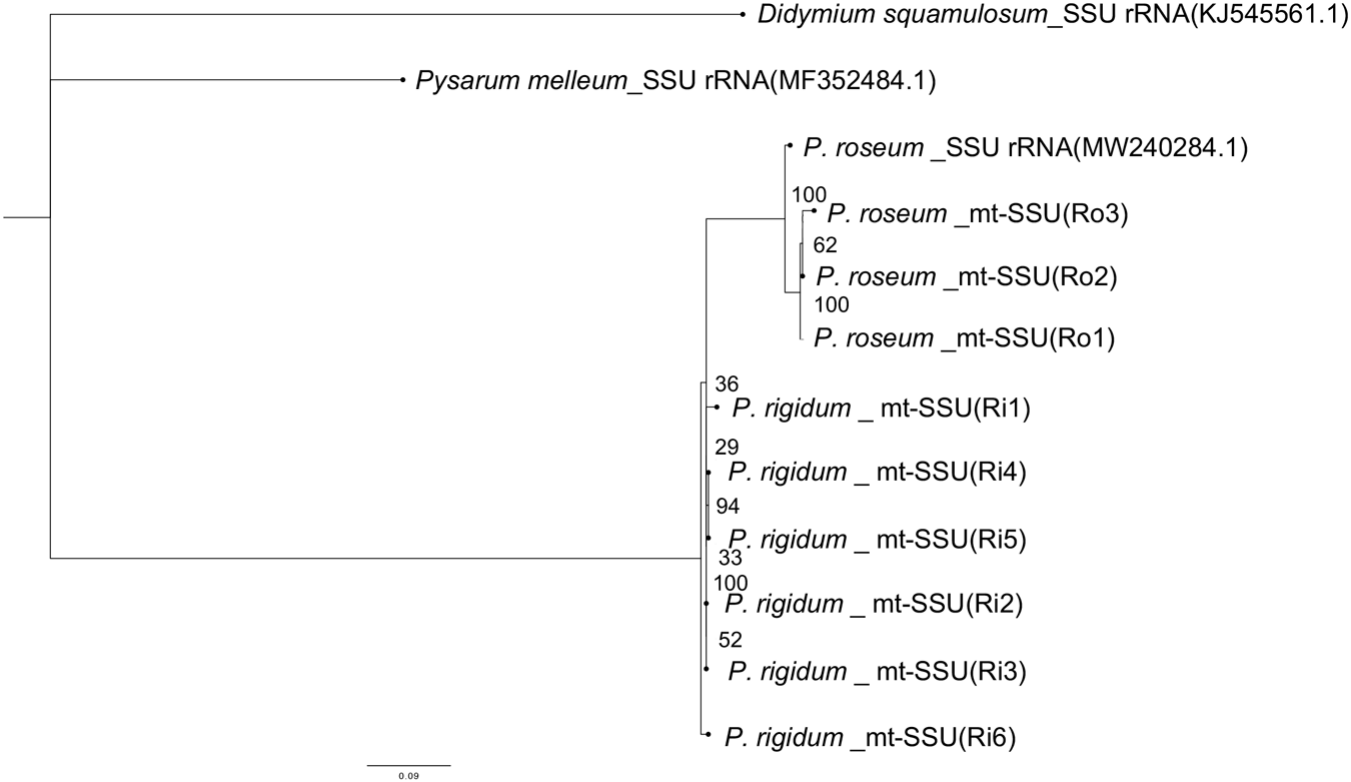
**Phylogenetic tree of the mt-SSU region.** Ri strains and Ro strains formed different clades. Additionally, Ro strains formed a clade identical to the known *P. roseum* clade.

Phylogenetic trees of all strains used in this study were constructed using the mt-SSU and 18S rRNA regions. The results showed that the Ri strains and Ro strains formed distinct clades, indicating that they are different species (Figs 2,3). Furthermore, the Ro strains formed the same clade as the existing *P. roseum* strains, confirming that they were *P. roseum*. The sequences of the Ri strains in the SSU rRNA region (ph series) were found to be identical to those of the existing *P. rigidum*, confirming them as *P. rigidum*.

### Intraspecies behavioral test

**Fig. 4.**
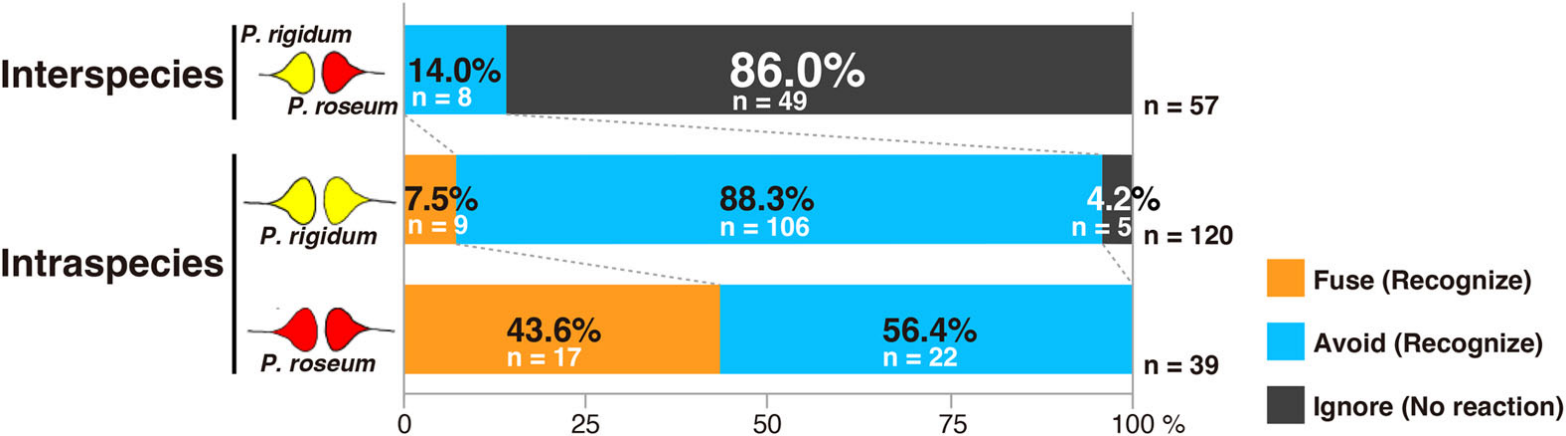
**Assessment results for behavioral tests between interspecies and intraspecies (*P. rigidum, P. roseum*)**. ‘Ignore’ indicates riding-over behavior. There was a predominant difference in the evaluation results for each combination, with a tendency to ignore the known allorecognition behavior between species (χ^2^=231.357, *P*<0.0001, Pearson's chi-square test). Here, *n* indicates the number of encounters between Plasmodium species in the behavioral tests conducted.

All of the anticipated encounter responses (fusion, avoidance, and no response) were observed in the intraspecific behavioral test, but their proportions varied widely (Fig. 3). Using *P. rigidum* as the intraspecies behavioral test, a total of 120 encounters were tested. In the intraspecies behavioral test using *P. rigidum*, behavioral experiments were conducted three times for each combination of the six strains. However, for the Ri1–Ri3 combination, due to the lower number of encounters, the experiments were conducted five times. The results showed that nine (7.50% of the total) exhibited fusion behavior. Avoidance behavior was observed in 106 cases (88.33% of the total). On the other hand, riding-over behavior (no response) was observed in only five cases (4.17% of the total) (Fig. 4). In the experiments with *P. roseum*, each combination was tested until more than 10 encounters were observed, resulting in a total of 39 encounters. Unlike in the experiments with *P. rigidum*, no behavior of riding over on the other individual was observed (Fig. 4). Seventeen (43.59%) of the total number of encounters showed fusion behavior, and 22 (56.41%) of the total number of encounters showed avoidance behavior (Fig. 4).

### Interspecies behavioral test

An interspecies recognition test was conducted by contrasting *P. rigidum* and *P. roseum*. A total of 10 experiments were conducted, resulting in 57 observed encounters. Interestingly, unlike intraspecies behavioral tests, fusion behavior was never observed. Avoidance behavior was exhibited in eight cases, or 14.04% of the total. The remaining 49 cases, or 85.96% of the total number of cases, exhibited riding-over behavior (Figs 1C and 4).

### Comparison of the percentage of allorecognition behaviors

The results of the behavioral tests (fusion, avoidance, and no response) of all 269 encounters were compared between/within different species/same species (*P. rigidum* and *P. roseum*), and significant differences were found (χ^2^=190.700, *P*<0.0001, Pearson's chi-square test) (Fig. 4). This result indicated that the slime molds tended to not exhibit fusion or avoidance, known as allorecognition behavior, with respect to other species. In other words, they did not specifically recognize other species and ride over them as obstacles. In all encounters, the plasmodium survived for more than 24 h after Myxomycetes settled on their behavior (fusion, avoidance, or no response).

## DISCUSSION

### Discovering a third behavior during plasmodium encounters

This study is the first to report that *P. roseum* has the ability to recognize self and others and to fuse, confirming that self- and other-recognizing behavior is common in the genus *Physarum*.

Furthermore, fusion and avoidance, which are allorecognition behaviors, were rarely observed between species (Fig. 4), even though the species belonged to the same genus, *Physarum*. Cross-genus verification of interspecies recognition has previously been performed; with *Physarum* and *Fuligo*, no cytoplasmic uptake upon contact has been detected (Zabka et al., 1962). The fact that no interspecies fusion behavior was observed in our experiments confirms the findings of previous studies.

In addition, a behavior that could be interpreted as completely ignoring the other individual was prominently observed at the interspecies level. It is a behavior that neither fuses nor avoids but rides over other individuals (Fig. 4). When the opponent is a homologous heterologous individual, if it is a target that cannot fuse, the behavior of avoiding the opponent has been observed in most cases (Masui et al., 2018). Avoidance behavior is a behavior in which the target is once recognized as a Plasmodium of a different individual and judged to be unable to become a single individual by fusion.

However, the behavior of ignoring is considered to be a failure to recognize the target as a Plasmodium of Myxomycetes in the first place. Therefore, this behavior can be defined as a third response that a plasmodium takes when it comes into contact with other individuals.

**Fig. 5.**
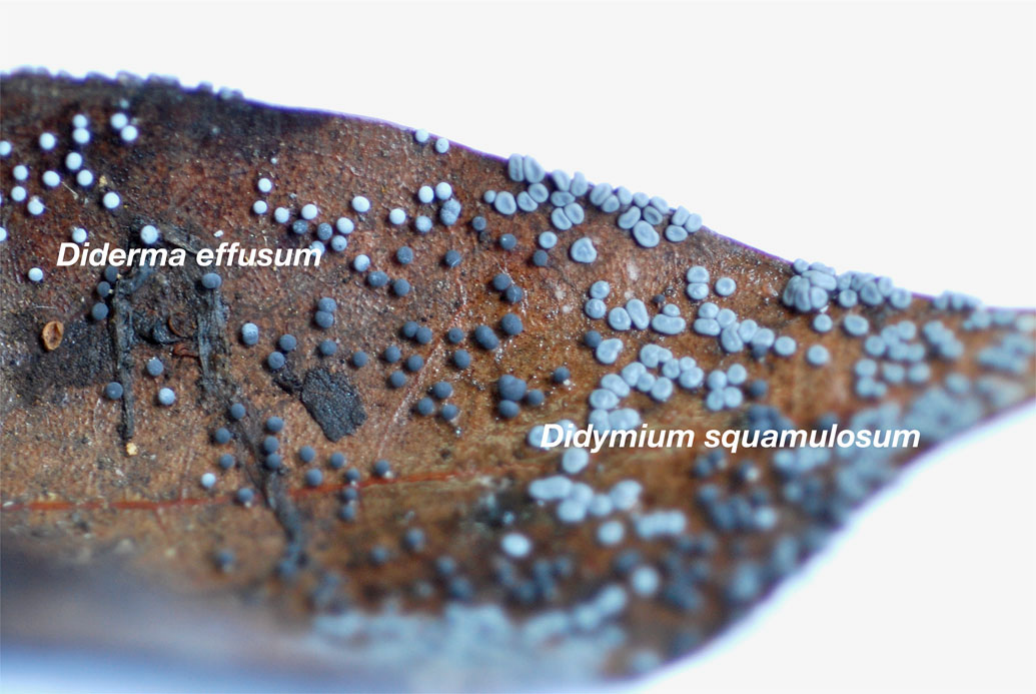
**Fruiting bodies are formed by two species of Myxomycetes next to each other.** The spherical shape is *Diderma effusum*. *Didymium squamulosum* is irregularly elliptical. As they both formed complete fruiting bodies, they did not fuse or attack each other in the plasmodium stage. Additionally, as neither fruiting body has completely desiccated, it is likely that they began forming their fruiting bodies almost simultaneously.

In addition to the lack of fusion, no behavior has been observed in previous studies or in this study in which heterophiles attacked each other upon contact. Examples of interspecies ignoring without mutual attack can also be seen in the field (Fig. 5). This means that Myxomycetes have the opportunity to contact many different species of Plasmodium. Therefore, contact with different Plasmodium species is not completely unexpected, and these species are not recognized as objects of fusion or avoidance and do not actually harm each other.

### Plasmodium's allorecognition behaviors can discriminate only between individuals of the same species

The behavior of ignoring observed in this experiment was almost never observed in intraspecies encounters and was therefore interspecies specific. This indicates that the allorecognition behaviors of Plasmodium occur only between individuals of the same species. Based on these results, it can be concluded that these behaviors are insufficient for avoiding plasmodium from other species and are suitable for searching for plasmodium from the same species, a group with the potential to fuse with each other.

This ‘ignoring’ behavior can be considered a failure of allorecognition for the following reasons: while individuals of the same species that cannot fuse typically exhibit clear avoidance behaviors; different species do not avoid each other but instead engage in ignoring behavior by riding over. It is highly unlikely that there is an advantage to performing this ignoring behavior specifically toward individuals of different species. Recognition involves both avoidance and fusion, where avoidance occurs when the plasmodium recognizes the other and moves away. In contrast, ignoring this context shows no observable response, which cannot be classified as a known allorecognition behavior. However, it is currently impossible to determine whether different species are truly outside the recognition range of the system due to the lack of understanding of the molecular mechanisms involved. This makes it difficult to consider the causes and significance of the 14% of avoidance cases that occurred in the interspecies test. Additionally, it is possible that the current species classification and the target of the recognition mechanism do not align. To understand these aspects, it is necessary to conduct genome-level comparisons between individuals and identify the specific mechanisms of the system. Additionally, the significance of this behavior remains in question. If the importance of this system is considered as above, it is necessary to consider its significance in the plasmodium resulting from behavior ‘fusion’. However, to date, the benefits of fusion have not been clearly demonstrated and have rarely been discussed in previous studies. Fusion between plasmodia is a phenomenon in which the membranes of the cells dissolve and the protoplasm mix with each other, but the nuclei do not fuse. It is therefore unlikely to contribute to increased genetic diversity in reproduction. One change that can be stated with certainty for plasmodium in fusion is the increase in the size of their own cells as a result of incorporating different individuals. The advantage of this approach is the formation of more fruiting bodies.

Myxomycetes form dozens to hundreds of fruiting bodies from a single plasmodium, in which tens of thousands to millions of spores are present (Schnittler and Tesmer, 2008). The number of spores produced per fruiting body varies among species to some extent, but the total number of spores formed by a single plasmodium is not species specific. Instead, the total number of spores formed by a single plasmodium depends on its size. This finding implies that there is a clear advantage for Plasmodium in increasing its cell size, which increases the chances of producing more offspring. Additionally, there are reports that a larger cell size is advantageous for spatial exploration (Tröger et al., 2024). However, the benefits and drawbacks of plasmodium cell size enlargement, including potential shifts in energy metabolism efficiency, were not examined in the present study due to inadequate knowledge.

However, significant intra- and interspecies variation in the response to contact can provide essential insights into inter-individual responses in Myxomycetes. For instance, previous research has indicated that the slime sheath produced by plasmodium is involved in allorecognition behavior. Signal substances for recognition have yet to be identified. The main component of the slime sheath has been identified as a glycoprotein, but the other components continue to be debated. The findings of the present study suggested that the signal substance used here is species specific. This is because allorecognition behaviors such as fusion and avoidance have only been observed on an intraspecies basis. Neither of the two species used in the verification showed any interspecies response, despite the similarity of the allorecognition behavior within the species. This finding suggested that the mechanisms of allorecognition are similar but that the signaling molecules involved are different.

Although this study allowed us to consider the ecological significance of the allorecognition behavior of Myxomycetes, the underlying molecular mechanisms are still unknown. We believe that clarifying the details of the loci that determine whether fusion is possible and the mechanisms that control this behavior will provide insight into the full range of interactions between individuals in Myxomycetes.

## MATERIALS AND METHODS

### Samples

Samples were collected at the Saitama Midorinomori Museum, Japan, using six independent *P. rigidum* strains (Ri1∼6 collected from August to September 2020) and three *P. roseum* strains sampled in the same field (Ro1 was obtained in September 2011, Ro2 and 3 were both obtained in August 2018).

The sampled plasmodia were preconditioned for more than 1 year under the following common culture conditions. Plasmodia were cultured on 2% agar medium in 9 cm diameter petri dishes and fed daily with water and oat flakes as nutrient sources. The temperature conditions for each species were maintained at 25°C for *P. rigidum* and 22°C for *P. roseum* using a cool incubator.

### Species identification

Species identification of the culture samples was performed using DNA barcoding targeting the SSU region and the construction of molecular phylogenetic trees. We used a total of three primers, two targeting the 18S rRNA region and one targeting the mt-SSU region, as referenced from previous studies (Table 1) (García-Martín et al., 2023; Kamono and Fukui, 2006; Wang et al., 2023).

**Table 1.**
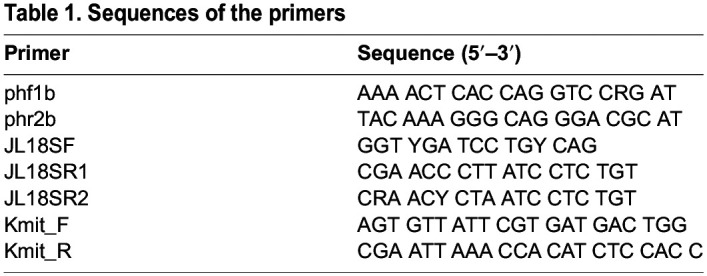
Sequences of the primers

The PCR conditions for each primer were as follows: ph series targeting the SSU rRNA region: 94°C, 1 min; [98°C, 10 s; 55°C, 15 s; 68°C, 1 min]×30; 68°C, 5 min (Kamono and Fukui, 2006).

JL18S series targeting the SSU rRNA region: 94°C, 3 min; [94°C, 20 s; 55°C, 30 s (decreasing at 1°C/cycle); 68°C, 50 s]×10; [94°C, 20 s; 55°C, 30 s; 68°C, 50 s]×30; 68°C, 10 min (Touchdown PCR) (Wang et al., 2023).

Kmit series targeting the mt-SSU region: 94°C, 1 min; [98°C, 10 s; 55°C, 1 min; 68°C, 1 min]×30; 68°C, 10 min (García-Martín et al., 2023).

The sequences were determined by DNA Sequencing Service (Eurofins Genomics Ltd.). The obtained sequences were aligned using the MAFFT online service (Katoh et al., 2019). Subsequently, molecular phylogenetic trees were constructed using the maximum likelihood method with IQ-TREE (version 2.1.4). For data obtained from primers that could not amplify all samples, sequence similarity searches were performed using NCBI BLAST. Additionally, the morphological characteristics of the fruiting bodies were used for supplementary identification (Schaap et al., 2016; Yamamoto, 1998).

### Behavioral test

Behavioral tests to verify Plasmodium allorecognition behavior were conducted based on a previously established method (Masui et al., 2018). Two plasmodia were placed 3 mm apart on 2% agar medium and recorded by time-lapse photography every minute until the two individuals fused or 2 days had elapsed. The experimental environment was kept at 25°C, with no direct light exposure, and the sample was watered with sterile water to prevent the surface of the medium from drying out. Both *P. rigidum* and *P. roseum* were used in intraspecies and interspecies behavioral tests. In each behavioral test, an ‘encounter’ was defined as behavior in which plasmodia stopped behaving within 3 mm of each other for at least 2 min or in which cell membranes made contact with each other and was assessed using the following framework.

Behavioral tests were qualitatively assessed using a tripartite framework comprising ‘fusion’, ‘avoidance’, and ‘no reaction (ignore)’. These indicators were meticulously documented and categorized up to the point of either ultimate fusion or total cellular separation. Encounter cases were classified using time-lapse photography. Fusion was defined as the fusion of cells with each other. Avoidance is defined as ceasing movement or a change in direction of travel without fusion occurring during observation. If the cells did not exhibit either of these two behaviors and exhibited the behavior of riding over on the other individual, it was classified as ‘no response (ignore)’. The above three indices were illustrated using stacked bar charts among and within different species (*P. rigidum* and *P. roseum*) for all encounters in the behavioral test. Pearson's chi-squared test was performed. JMP (version 14.3) was used for the statistical analysis.
